# Switch from reference etanercept to SDZ ETN, an etanercept biosimilar, does not impact efficacy, safety, and immunogenicity of etanercept in patients with moderate-to-severe rheumatoid arthritis: 48-week results from the phase III, randomized, double-blind EQUIRA study

**DOI:** 10.1186/s13075-019-1907-x

**Published:** 2019-05-28

**Authors:** Janusz Jaworski, Marco Matucci-Cerinic, Hendrik Schulze-Koops, Maya H. Buch, Eugeniusz J. Kucharz, Yannick Allanore, Arthur Kavanaugh, Philip Young, Goran Babic

**Affiliations:** 1 Reumatika–Centrum Reumatologii, 02-691 Warsaw, Poland; 2 Department of Experimental and Clinical Medicine, Division of Rheumatology AOUC, University of Florence, Florence, Italy; 3 Division of Rheumatology and Clinical Immunology, Department of Internal Medicine IV, Ludwig-Maximilians-University, Munich, Germany; 4 Leeds Institute of Rheumatic & Musculoskeletal Medicine, University of Leeds & NIHR Leeds Biomedical Research Centre, Leeds, UK; 5 Department of Internal Medicine, Rheumatology and Clinical Immunology, Medical University of Silesia, Katowice, Poland; 6 Cochin Hospital, Rheumatology A department, Paris Descartes University, Paris, France; 7 UC San Diego School of Medicine, La Jolla, CA USA; 8 Hexal AG, a Sandoz company, Holzkirchen, Germany

**Keywords:** Biosimilar, Etanercept, Rheumatoid arthritis, SDZ ETN, Switch, Tumor necrosis factor inhibitor

## Abstract

**Background:**

Sandoz etanercept (SDZ ETN; GP2015) is an etanercept biosimilar with equivalent efficacy and comparable safety and immunogenicity to reference etanercept (ETN) in patients with moderate-to-severe chronic plaque-type psoriasis.

**Methods:**

EQUIRA was a phase III, double-blind study conducted in patients with moderate-to-severe rheumatoid arthritis and inadequate response to disease-modifying anti-rheumatic drugs. Eligible patients were randomized 1:1 to receive subcutaneous 50 mg SDZ ETN or ETN, once-weekly, for 24 weeks. At week 24, patients with at least moderate EULAR response in the SDZ ETN group continued SDZ ETN treatment, and those in the ETN group were switched to receive 50 mg SDZ ETN, for up to 48 weeks. Patients received concomitant methotrexate at a stable dose (10–25 mg/week) and folic acid (≥ 5 mg/week). Equivalence between SDZ ETN and ETN for change from baseline in disease activity score including 28 joint count C-reactive protein (DAS28-CRP) at week 24 (primary endpoint) and comparable safety and immunogenicity profile of SDZ ETN and ETN have previously been demonstrated at week 24. Herein, we present the 48-week results of the study after a single switch from ETN to its biosimilar at week 24.

**Results:**

The least squares mean (standard error) change in DAS28-CRP from baseline up to week 48 was comparable between “continued SDZ ETN” (− 2.90 [0.12], *n* = 148) and “switched to SDZ ETN” (− 2.78 [0.13], *n* = 131) groups. The proportion of patients achieving EULAR good/moderate responses based on DAS28-erythrocyte sedimentation rate and ACR20/50/70 response rates were comparable between the two groups. The proportion of patients with at least one treatment-emergent adverse event was 42.9% in the “continued SDZ ETN” and 38.0% in the “switched to SDZ ETN” groups. Serious adverse events occurred in 4 patients in each of the two groups. After week 24, none of the patients in the switched group developed anti-drug antibodies (ADAs), while 4 patients in the continued SDZ ETN group had single-event, very low titer, non-neutralizing ADAs detected.

**Conclusions:**

The 48-week results from the EQUIRA study demonstrate that switch from ETN to SDZ ETN in patients with moderate-to-severe rheumatoid arthritis does not impact the efficacy, safety, or immunogenicity of etanercept.

**Trial registration:**

EudraCT number 2012-002009-23, Registered 19 April 2012—prospectively registered.

**Electronic supplementary material:**

The online version of this article (10.1186/s13075-019-1907-x) contains supplementary material, which is available to authorized users.

## Background

Etanercept, a tumor necrosis factor inhibitor, has been used successfully for the treatment of multiple immune-mediated inflammatory diseases including moderate-to-severe rheumatoid arthritis (RA) [1].

Sandoz etanercept (SDZ ETN; development name GP2015, Erelzi® [Sandoz Inc., Princeton, NJ 08540]) is an etanercept biosimilar. Pharmacokinetic equivalence and comparable safety for SDZ ETN and reference etanercept (ETN; Enbrel® [European Union-authorized]) was demonstrated in a phase I study in healthy subjects [2]. The phase III EGALITY study demonstrated equivalent efficacy and comparable safety and immunogenicity of SDZ ETN and ETN in patients with moderate-to-severe chronic plaque-type psoriasis [3].

The randomized, double-blind, EQUIRA study demonstrated similar efficacy and comparable safety and immunogenicity profile of SDZ ETN to ETN at week 24 in patients with moderate-to-severe RA who had an inadequate response to either conventional synthetic (cs) and/or biologic (b) disease-modifying anti-rheumatic drugs (DMARDs) [4]. Herein, we present the 48-week results from the study on the effects of a single switch between ETN and SDZ ETN at week 24 on efficacy, safety, and immunogenicity of etanercept.

## Methods

### Study population

Patients, aged ≥ 18 years, were included if they met the following criteria: (1) RA diagnosed according to the American College of Rheumatology (ACR) 1987 or ACR/European League Against Rheumatism (EULAR) 2010 criteria [5] for ≥ 6 months before baseline; (2) active disease defined as disease activity score including 28 joint count (DAS28)-C-reactive protein (CRP) ≥ 3.2; (3) CRP > 5 mg/L or erythrocyte sedimentation rate (ESR) ≥ 28 mm/h; (4) inadequate clinical response to methotrexate (MTX) at a dose of 10–25 mg/week after optimal dose escalation according to local standards (those who had failed a csDMARD other than MTX, and any other csDMARD used in combination with MTX prior to baseline, were allowed after an appropriate wash-out period of 4 weeks); (5) MTX therapy for ≥ 3 months and on a stable dose for ≥ 28 days prior to baseline; (6) stable dose of folic acid (≥ 5 mg per week) for ≥ 28 days before baseline.

The key exclusion criteria included (1) any previous exposure to ETN; (2) treatment with any other bDMARD therapy for RA, including tumor necrosis factor (TNF) inhibitors, anti-CD20, immune-modulator drug(s), other investigational drug(s), and/or device(s) within 3 months or 5 half-lives at the time of enrollment, whichever was longer; (3) previous use of > 2 bDMARDs (patients in whom bDMARDs were efficacious but withdrawn because of reasons other than efficacy failure or safety issues were not excluded); (4) functional status class IV according to the ACR 1991 revised criteria [6]; (5) systemic manifestations of RA, with the exception of Sjögren’s syndrome; (6) any active inflammatory or autoimmune diseases other than RA; and (7) tuberculosis or latent tuberculosis detected by imaging and/or by the QuantiFERON®-TB Gold test at screening.

### Study design

The EQUIRA study was conducted from 27 November 2015 to 12 June 2017 at 83 study centers across 16 countries (NCT02638259). Eligible patients were randomized 1:1 to self-administer 50 mg SDZ ETN or ETN (Enbrel® [European Union-authorized]), provided as pre-filled syringes, subcutaneously, once-weekly, for 24 weeks (treatment period 1 [TP1]). At week 24, patients achieving at least a moderate treatment response according to EULAR response criteria [7] in the SDZ ETN group continued SDZ ETN (defined as “continued SDZ ETN” group), and those in the ETN group were switched to SDZ ETN (defined as “switched to SDZ ETN” group), for up to 48 weeks (TP2). The initial randomization schedule and blinding have been described previously [4].

This study was conducted in accordance with the ethical principles derived from the Declaration of Helsinki and International Conference on Harmonization Good Clinical Practices and in compliance with local regulatory requirements. The study protocol was approved by the Independent Ethics Committee or Institutional Review Board for each center. All patients provided written informed consent before entering the study.

### Study assessments

The primary endpoint of the study was the change from baseline in DAS28-CRP up to week 24. The secondary endpoints, assessed up to week 24 and week 48 included (i) change from baseline in DAS28-CRP scores [8]; (ii) proportion of patients achieving good and moderate EULAR response based on DAS28-ESR [9]; (iii) proportion of patients achieving an improvement in the ACR20/50/70 response rates; (iv) physical function assessed by the health assessment questionnaire disability index (HAQ-DI) score [10, 11], and proportion of patients achieving HAQ index in normal range (≤ 0.5); and (v) impact of fatigue on patients assessed by the Functional Assessment of Chronic Illness Therapy (FACIT) fatigue scale [12, 13].

Safety assessments included evaluation of the adverse events (AEs) as well as the local tolerability of injection sites of both medications as assessed by the investigator during the study. Immunogenicity assessment included analysis of anti-drug antibodies (ADAs) up to 48 weeks using a validated screening, confirmatory, and titer determination electrochemiluminescence bridging assay [14].

### Statistical analysis

The sample size determination has been described previously [4]. The TP2 full analysis set (FAS) consisted of all patients who continued to TP2 and had at least one study assessment documented in TP2. The TP2 per-protocol set (PPS) consisted of all patients completing the study until week 48 without major protocol deviations (major protocol deviations are listed in Additional file 1**:** Table S1); patients who prematurely withdrew from the study were also not included, although this was not a protocol deviation. The TP2 safety set (SAF) consisted of all patients who received at least one dose of study treatment during TP2. All efficacy analyses were performed on the TP2 PPS and repeated on the TP2 FAS. TP2 SAF was used for the safety summaries in TP2.

During TP2, the “continued SDZ ETN” group was compared with the “switched to SDZ ETN” group. A repeated measures analysis of (co)variance with treatment and time as factors up to week 48 was performed for DAS28-CRP change from baseline. Change from baseline in DAS28-CRP was estimated using values up to week 48 from the same mixed-model repeated measures analysis used to analyze the primary endpoint.

## Results

### Patient disposition and baseline characteristics

**Fig. 1:**
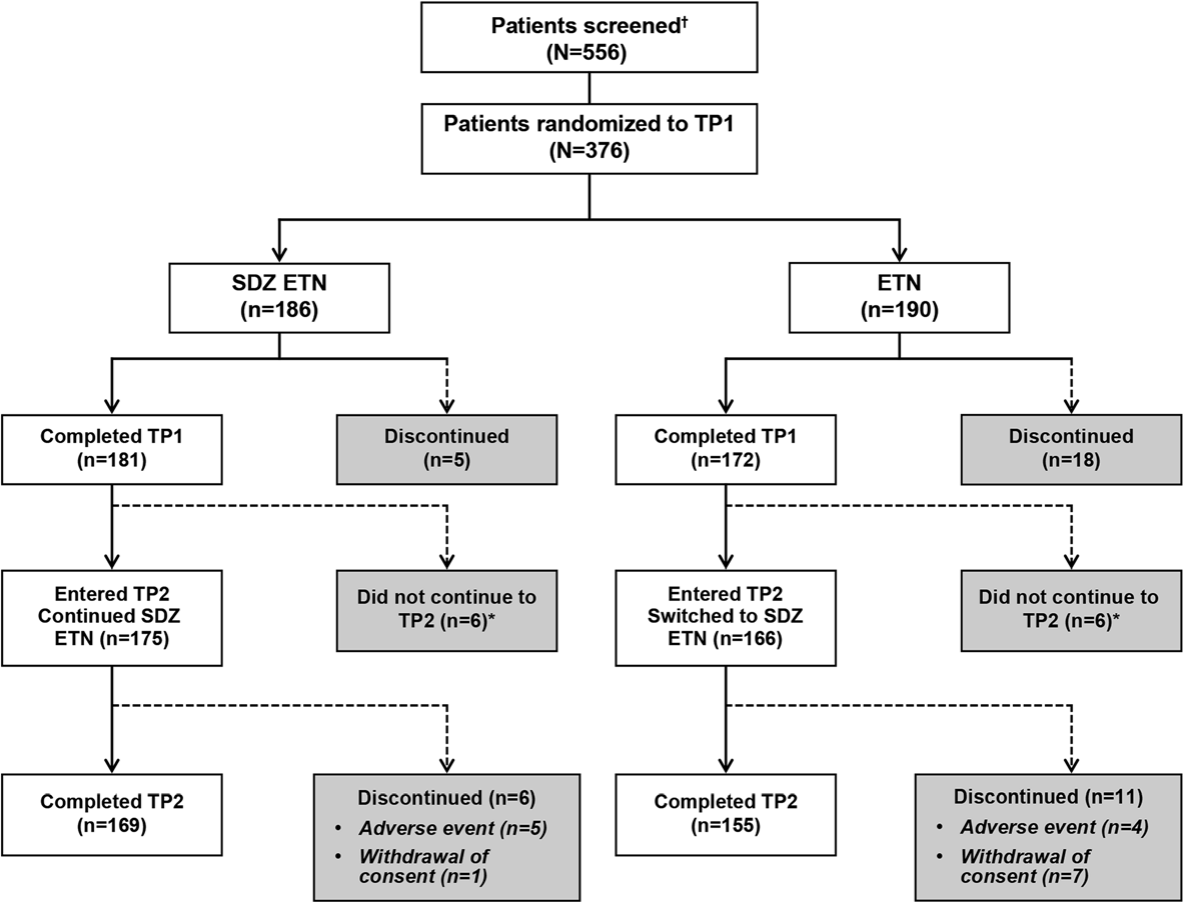
Patient disposition. ^†^The primary reason for not completing screening phase included: inclusion/exclusion criteria not fulfilled (*n* = 169), withdrawal of consent (*n* = 12), adverse event (*n* = 1), lost to follow-up (*n* = 1), and other (*n* = 8); *Four patients in each treatment group were not eligible for TP2; 2 patients in each treatment group were eligible but did not enter TP2; ETN, reference etanercept; SDZ ETN, Sandoz etanercept; TP, treatment period

Of the 376 patients randomized in the study, 353 (SDZ ETN, *n* = 181; ETN, *n* = 172) completed TP1. A total of 341 patients entered TP2, of whom 324 completed TP2 (Fig. 1). The reasons for discontinuation during TP2 were AEs (SDZ ETN, *n* = 5 [2.9%]; ETN, *n* = 4 [2.4%]) and withdrawal of patient consent (SDZ ETN, *n* = 1 [0.6%]; ETN, *n* = 7 [4.2%]). All patients who entered TP2 were included in the TP2 FAS and TP2 SAF. The TP2 PPS included 279 patients (148 [84.6%] and 131 [78.9%] patients in the SDZ ETN and ETN groups, respectively).

In the overall population who entered TP2, mean (SD) age was 53.7 (12.0) years, most (80.4%) patients were in the 18–64 age category, and majority (82.1%) were females. The mean (SD) duration of RA was 8.4 (7.6) years and most (71.3%) patients were categorized as functional RA class II. The most common prior medications other than MTX and folic acid were methylprednisolone (28.2%), meloxicam (15.2%), and ibuprofen (7.6%). Patient demographics and baseline characteristics were well-balanced and comparable between TP2 treatment groups (Table 1).

**Table 1:** Baseline demographics and disease characteristics (TP2 full analysis set)

Characteristics	Continued SDZ ETN (*n* = 175)	Switched to SDZ ETN (*n* = 166)
Age (years)	55.1 (10.99)	52.2 (12.84)
Female, *n* (%)	149 (85.1)	131 (78.9)
Race,^a^ *n* (%)		
Caucasian	169 (96.6)	164 (98.8)
Functional RA status, *n* (%)		
Class I	20 (11.4)	25 (15.1)
Class II	122 (69.7)	121 (72.9)
Class III	33 (18.9)	20 (12.0)
DAS28-CRP	5.42 (0.92)	5.54 (0.78)
DAS28-ESR	6.34 (0.88)	6.42 (0.76)
Tender 28 joint count	14.1 (6.21)	14.5 (5.57)
Swollen 28 joint count	10.6 (5.22)	11.0 (5.39)
C-reactive protein (mg/L)	12.0 (21.63)	11.3 (16.34)
HAQ-DI score	1.45 (0.55)	1.47 (0.56)
FACIT-fatigue score	26.82 (9.55)	25.32 (10.14)
Duration of rheumatoid arthritis (years)	8.75 (8.22)	8.11 (6.93)
Rheumatoid factor, positive,^b^ *n* (%)	130 (74.30)	118 (71.10)
Anti-CCP, positive, ^b^ *n* (%)	138 (78.90)	119 (71.70)
Prior therapy,^c^ *n* (%)		
MTX only	53 (30.3)	46 (27.7)
MTX + any DMARDs	68 (38.9)	69 (41.6)
MTX + any anti-TNF	30 (17.1)	28 (16.9)
MTX + any other biologic	24 (13.7)	23 (13.9)
Previous DMARDs used, *n* (%)		
1	53 (30.3)	46 (27.7)
2	69 (39.4)	62 (37.3)
3	34 (19.4)	39 (23.5)
4 or more	19 (10.9)	19 (11.4)
MTX dose (mg/week)	16.0 (4.9)	17.0 (4.7)
Duration of MTX (months)	56.3 (49.9)	59.3 (52.4)

Values are mean (SD) unless stated otherwise

*CCP* cyclic citrullinated peptide, *DAS28-CRP* disease activity score 28-joint count, C-reactive protein, *DMARDs* disease-modifying anti-rheumatic drugs, *ESR* erythrocyte sedimentation rate, *ETN* reference etanercept, *FACIT* Functional Assessment of Chronic Illness Therapy, *HAQ-DI* Health assessment questionnaire disability index, *MTX* methotrexate, *RA* rheumatoid arthritis, *SDZ ETN* Sandoz etanercept, *SD* standard deviation, *TNF* tumor necrosis factor, *TP2* treatment period 2

^a^Other race categories in “continued SDZ ETN” group included Black or African American (*n* = 5), and American Indian or Alaska Native (*n* = 1), and in “switched to SDZ ETN” group included Asian (*n* = 1) and American Indian or Alaska Native (*n* = 1)

^b^Rheumatoid factor ≤ 10 UI/mL and anti-CCP < 17 U/mL are considered negative

^c^Prior therapy strata is arranged according to the hierarchy

### Efficacy

The primary objective of the study to demonstrate that therapeutic equivalence between SDZ ETN and ETN was met, as the 95% confidence interval (CI) for the least squares mean difference between SDZ ETN and ETN for change from baseline in DAS28-CRP at week 24 was contained within the pre-specified equivalence margin of [− 0.6, 0.6] [4].

### Efficacy up to week 48

**Fig. 2:**
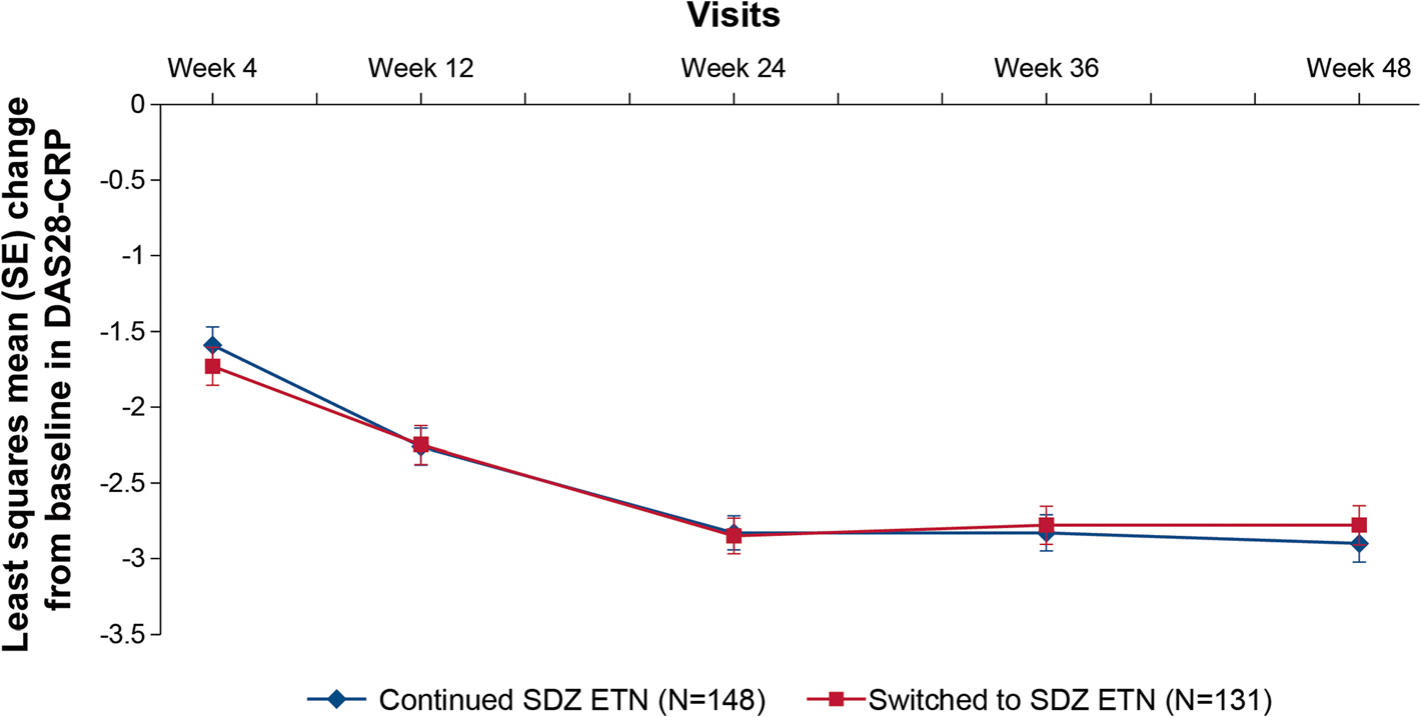
DAS28-CRP change from baseline up to week 48 (TP2 per-protocol set). Analyzed using a repeated measures analysis of (co)variance with treatment and time as factors up to week 48. TP2 PPS comprised all patients completing the study until week 48 without major protocol deviations. CRP, C-reactive protein; DAS28, disease activity score including 28 joint count; PPS, per-protocol set; SE, standard error; TP, treatment period

**Fig. 3:**
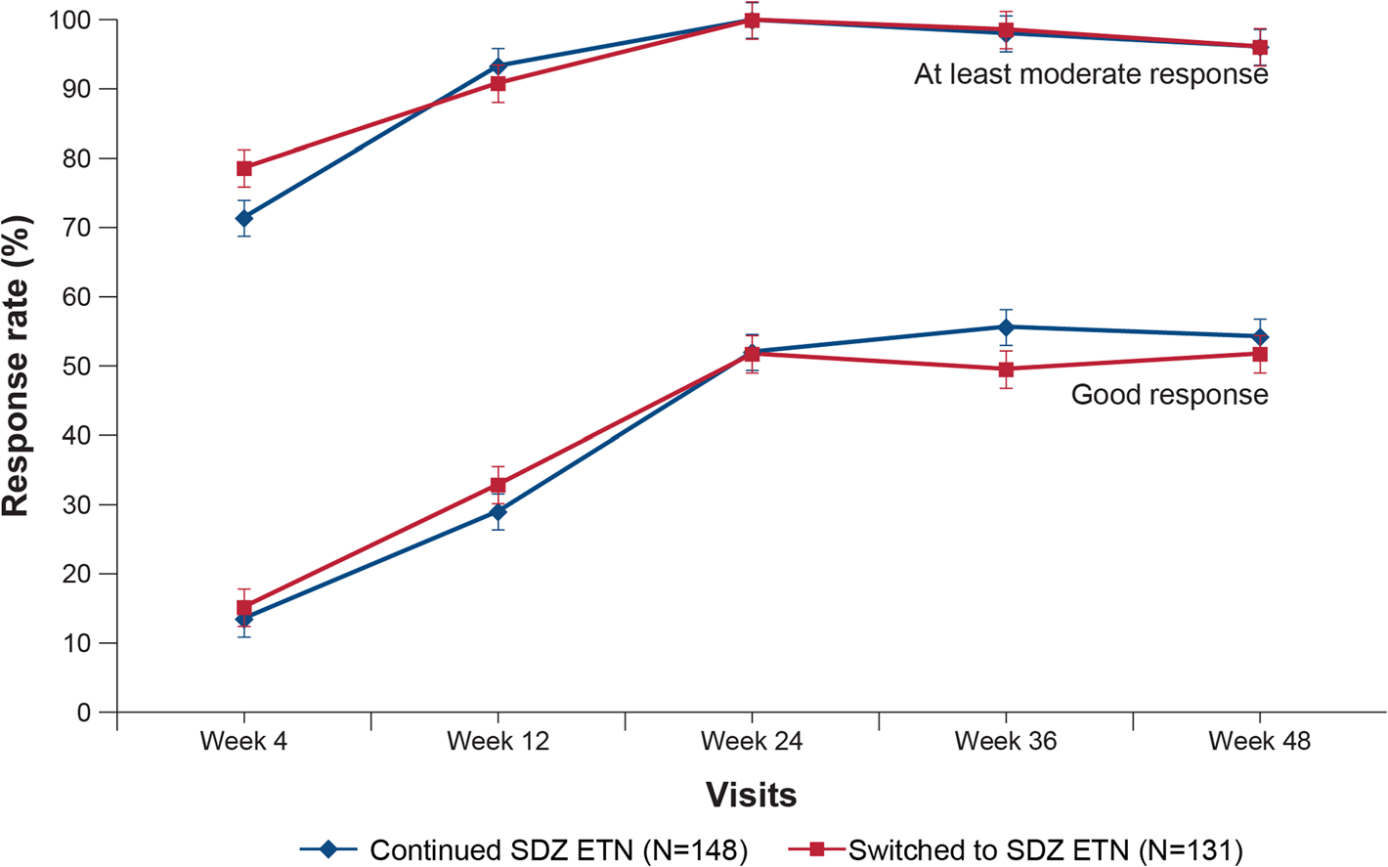
EULAR good and moderate responses up to week 48 (TP2 per-protocol set). Response rate of at least moderate response = good response + moderate response; EULAR good response: DAS28 ≤ 3.2 and DAS28 improvement from baseline > 1.2; EULAR moderate response: DAS28 ≤ 3.2 and DAS28 improvement > 0.6 and ≤ 1.2, or DAS28 >3.2 and ≤5.1 and DAS28 improvement > 0.6 or DAS28 >5.1 but DAS28 improvement > 1.2. TP2 PPS comprised all patients completing the study until week 48 without major protocol deviations. EULAR, European League Against Rheumatism; PPS, per-protocol set; TP, treatment period

**Fig. 4:**
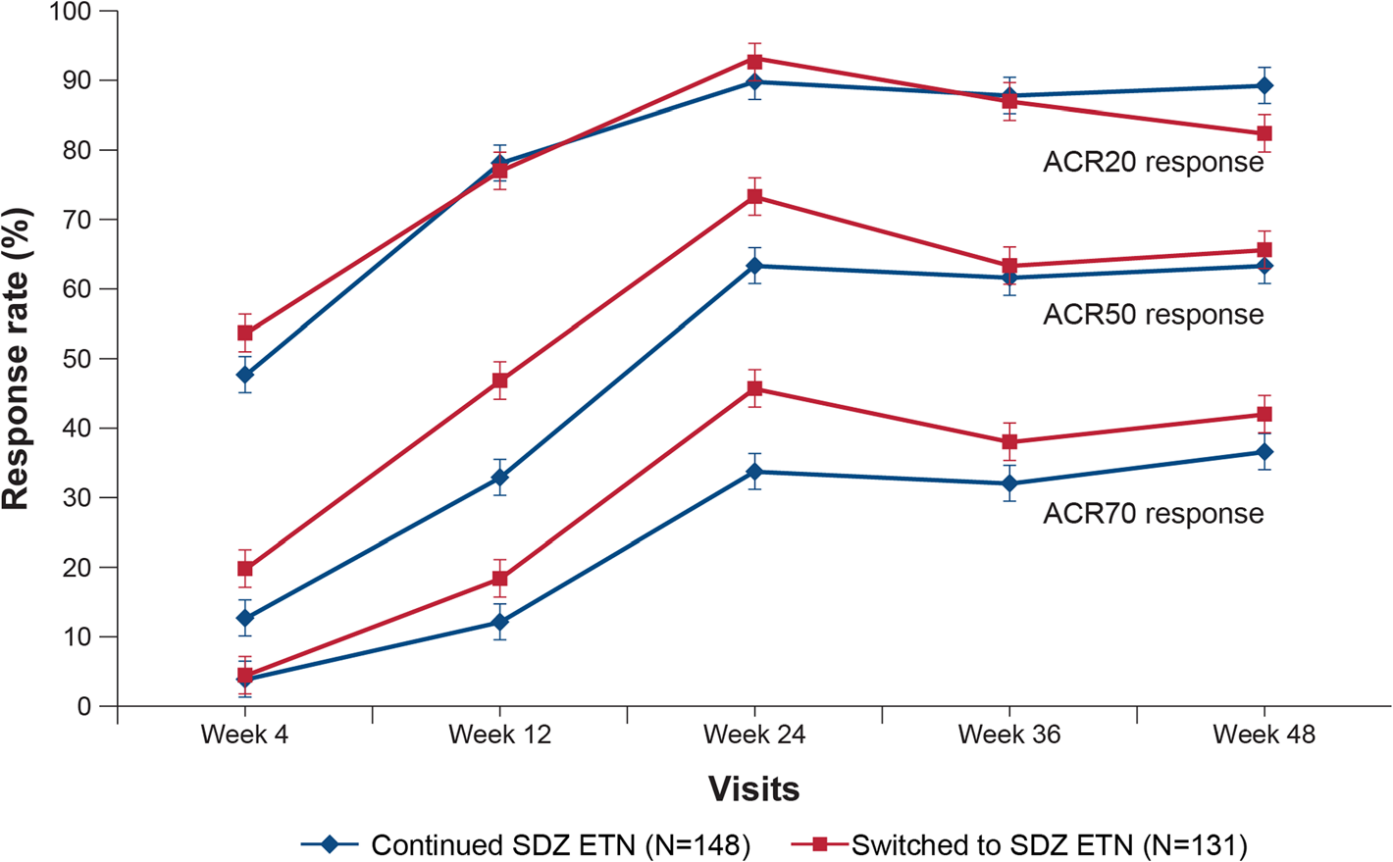
ACR20, ACR50, and ACR70 response rates up to week 48 (TP2 per-protocol set). TP2 PPS comprised of all patients completing the study until week 48 without major protocol deviations. ACR, American College of Rheumatology; PPS, per-protocol set; TP, treatment period

The least squares mean (SE) DAS28-CRP change from baseline to week 48 was comparable between “continued SDZ ETN” (− 2.90 [0.12]) and “switched to SDZ ETN” (− 2.78 [0.130]) groups (Fig. 2). The proportion of patients achieving EULAR “good” and “moderate” responses based on DAS28-ESR was similar between “continued SDZ ETN” and “switched to SDZ ETN” groups (week 48: “continued SDZ ETN” group vs “switched to SDZ ETN” group: EULAR good response, 54.4% vs 51.9%; EULAR moderate response, 41.5% vs 44.2%; Fig. 3). The proportion of patients achieving ACR20, ACR50, and ACR70 response was generally comparable between “continued SDZ ETN” and “switched to SDZ ETN” groups; ACR50 and ACR70 response rates were numerically higher in the “switched to SDZ ETN” group at all time-points, but not clinically relevant (Fig. 4). At week 48, ACR20, ACR50, and ACR70 response rates were 89.1%, 63.3%, and 36.7%, respectively, in the “continued SDZ ETN” group and 82.4%, 65.6%, and 42.0% in the “switched to SDZ ETN” group.

At week 48, the mean (SD) change from baseline in HAQ-DI score was − 0.62 (0.55) in the “continued SDZ ETN” group and − 0.66 (0.55) in the “switched to SDZ ETN” group, and the mean (SD) change from baseline in FACIT-fatigue score was 11.6 (9.7) in the “continued SDZ ETN” group and 10.6 (9.7) in the “switched to SDZ ETN” group (Additional file 1: Table S2). The proportion of patients achieving HAQ-DI in normal range (≤ 0.5) up to week 48 was comparable between “continued SDZ ETN” group (34.7%) and “switched to SDZ ETN” group (39.5%).

### Safety during TP2

The median (min, max) duration of exposure to study drug was similar in both treatment groups (“continued SDZ ETN”, 162 days [8–174]; “switched to SDZ ETN”, 162 days [1–169]). The proportion of patients with at least one treatment-emergent AE (TEAE) was comparable in the “continued SDZ ETN” (*n* = 75, 42.9%) and “switched to SDZ ETN” (*n* = 63, 38.0%) groups. Nasopharyngitis was the TEAE with highest incidence in the “continued SDZ ETN” and “switched to SDZ ETN” groups (7.4% vs 5.4%) followed by upper respiratory tract infection (5.1% vs 5.4%; Table 2). Injection site reaction as a TEAE was only reported in the “switched to SDZ ETN” group (3.6%; Table 2), and all were considered by the investigators to be treatment-related.

**Table 2:** Any TEAEs and treatment-related TEAEs with a ≥ 2% incidence in any of the treatment groups (TP2 safety set)

Preferred term	Continued SDZ ETN (*n* = 175) *n* (%)	Switched to SDZ ETN (*n* = 166) *n* (%)
TEAEs		
Nasopharyngitis	13 (7.4)	9 (5.4)
Upper respiratory tract infection	9 (5.1)	9 (5.4)
Urinary tract infection	7 (4.0)	2 (1.2)
Alanine aminotransferase increased	4 (2.3)	6 (3.6)
Injection site reaction	0	6 (3.6)
Headache	0	4 (2.4)
Treatment-related TEAEs		
Nasopharyngitis	5 (2.9)	0
Injection site reaction	0	6 (3.6)

A patient with multiple occurrences of event within the same system organ class or preferred term under one treatment is counted only once. TEAEs are events started after the first dose of study treatment and before study discontinuation or 30 days after last dose, whichever occurs later. Events are listed by descending order of occurrence in the “continued SDZ ETN” group

Adverse event terms are coded using MedDRA version 19.1

*ETN* reference etanercept, *MedDRA* medical dictionary for regulatory activities, *SDZ ETN* Sandoz etanercept, *TEAE* treatment-emergent adverse event

No deaths were reported. The proportion of patients with at least one serious adverse event (SAE) was low and comparable between the two treatment groups (*n* = 4 in each group): “continued SDZ ETN” group: pneumonia, salivary gland cyst, tibia fracture and cystitis hemorrhagic in 1 patient [0.6%] each; “switched to SDZ ETN” group: osteomyelitis, breast cancer, colon adenoma, cardiac failure, and acute cholecystitis in 1 patient [0.6%] each. The SAEs of acute cholecystitis and osteomyelitis reported in the “switched to SDZ ETN” group were suspected to be related to the study drug by the investigator. Treatment-related TEAEs occurred in 23 (13.1%) patients in the “continued SDZ ETN” group and in 19 (11.4%) patients in the “switched to SDZ ETN” group. The treatment-related TEAEs with the highest incidence were nasopharyngitis (2.9%) in the “continued SDZ ETN” group" and injection site reactions (3.6%) in the “switched to SDZ ETN” group (Table 2).

Four (2.3%) patients in the “continued SDZ ETN” group (benign breast neoplasm, genitourinary tract neoplasm, pneumonia, cystitis hemorrhagic; 1 patient [0.6%] each) and 4 (2.4%) patients in the “switched to SDZ ETN” group (breast cancer, injection site reaction and alanine aminotransferase increase, acute cholecystitis, skin hyperpigmentation; 1 patient [0.6%] each) discontinued due to TEAEs. TEAEs of special interest were reported in 9 (5.1%) patients in the “continued SDZ ETN” group and 12 (7.2%) in the “switched to SDZ ETN” group (Additional file 1: Table S3).

### Immunogenicity

Over 48 weeks, the proportion of ADA positive patients was small (< 3%) and comparable in the SDZ ETN/continued SDZ ETN groups and ETN/switched to SDZ ETN groups. After week 24, none of the patients in the switched group developed ADAs, while 4 patients in the continued SDZ ETN group had single-event, very low titer, non-neutralizing ADAs detected (Additional file 1: Table S4).

## Discussion

The advent of biosimilars has increased the possibility for switching between the reference medicine and its biosimilars, and this process is being evaluated in several countries [15–18]. The 48-week results from the EQUIRA study demonstrates that switching patients from ETN to SDZ ETN did not impact the efficacy, safety, or immunogenicity of etanercept in patients with moderate-to-severe RA. All efficacy parameters including DAS28-CRP change from baseline, EULAR good/moderate response rates based on DAS28-ESR, ACR 20/50/70 response rates and all other efficacy parameters, assessed up to 48 weeks, were comparable between the two treatment groups. Although numerical differences between the two groups were observed in the ACR response rates at week 48, these differences were not clinically relevant.

Sandoz etanercept was well tolerated, and no new or unexpected safety signals were detected in this study. Overall, the incidence of TEAEs and SAEs up to week 48 were comparable between the “continued SDZ ETN” and “switched to SDZ ETN” treatment groups.

For biosimilars, immunogenicity is an important aspect for the evaluation of clinical comparability [19]. In this study, a validated state-of-the-art technique comprising a high sensitivity and drug tolerance, which enables the detection of low titer and transient ADAs was used for the detection of ADAs. A false-positive rate for confirmatory assay of 1% was applied, as recommended recently [20], instead of the commonly used 0.1% rate [21]. Previous reports have shown that applying a sensitive as well as a drug-tolerant assay may lead to a higher reported incidence of ADA compared with historical data [14].

During TP2, 4 (2.4%) patients in the continued SDZ ETN group had single event. All the measured ADAs were of low titer, near the detection limit of the applied highly sensitive method. The detected ADAs were transient and non-neutralizing, which is in line with published data showing that ETN has low immunogenicity, and ADAs, if any, appear most prominently at week 4 and disappear afterwards [22]. In addition, the ADAs detected in this study were not clinically relevant as no correlation was observed between the immunogenicity outcome and patients’ efficacy and safety.

The results are consistent with the findings from the EGALITY study in patients with plaque-type psoriasis, which also showed that switching between ETN and SDZ ETN does not have an impact on efficacy, safety, or immunogenicity of etanercept [23]. In addition, the results also support data on switching from other clinical trials in different indications [3, 15, 17, 23]. Future patient registry studies would help to further confirm the effect of switching on long-term efficacy, safety, and immunogenicity.

## Conclusions

The 48-week results from the EQUIRA study demonstrate that switch from ETN to the biosimilar SDZ ETN in patients with moderate-to-severe RA did not impact the efficacy, safety, or immunogenicity of a continuous etanercept therapy.

## Additional file

Additional file 1: **Table S1.** Protocol deviations defined as major by category. **Table S2.** HAQ-DI and FACIT-fatigue scores over 48 weeks (TP2 per-protocol set). **Table S3.** TEAEs of special interest (TP2 safety set). **Table S4.** Summary of anti-drug antibodies up to week 48 using a 1% false-positive cut-point (Safety set). (DOCX 37 kb)

## Abbreviations

ACR: American College of Rheumatology
ADAs: Anti-drug antibodies
AEs: Adverse event
CRP: C-reactive protein
DAS28: Disease activity score including 28 joint count
DMARDs: Disease-modifying anti-rheumatic drugs
ESR: Erythrocyte sedimentation rate
EULAR: European League Against Rheumatism
FACIT: Functional Assessment of Chronic Illness Therapy
FAS: Full analysis set
HAQ-DI: Health assessment questionnaire disability index
MedDRA: Medical dictionary for regulatory activities
PPS: Per-protocol set
RA: Rheumatoid arthritis
SAE: Serious adverse event
SDZ ETN: Sandoz etanercept
TEAE: Treatment-emergent adverse event
TP: Treatment period
