# Supplementary material for: Switch from reference etanercept to SDZ ETN, an etanercept biosimilar, does not impact efficacy, safety, and immunogenicity of etanercept in patients with moderate-to-severe rheumatoid arthritis: 48-week results from the phase III, randomized, double-blind EQUIRA study
Source: Arthritis Res Ther. 2019 May 28;21:130. doi: 10.1186/s13075-019-1907-x (PMC6540397; doi:10.1186/s13075-019-1907-x)
Supplement: Supplementary file 1 — Table S1. Protocol deviations defined as major by category. Table S2. HAQ-DI and FACIT-fatigue scores over 48 weeks (TP2 per-protocol set). Table S3. TEAEs of special interest (TP2 safety set). Table S4. Summary of anti-drug antibodies up to week 48 using a 1% false-positive cut-point (Safety set). (DOCX 37 kb) [file 13075_2019_1907_MOESM1_ESM.docx]

**Additional file 1**

**Switch from reference etanercept to SDZ ETN, an etanercept biosimilar, does not impact efficacy, safety and immunogenicity of etanercept in patients with moderate to severe rheumatoid arthritis: 48-week results from the phase III, randomized, double-blind EQUIRA study**

Janusz Jaworski,^1^ Marco Matucci-Cerinic,^2^ Hendrik Schulze-Koops,^3^ Maya H. Buch,^4^ Eugeniusz J. Kucharz,^5^ Yannick Allanore,^6^ Arthur Kavanaugh,^7^ Philip Young,^8^ Goran Babic^8^

^1^Reumatika–Centrum Reumatologii, Warsaw, Poland (januszjaworski@ymail.com);

^2^Department of Experimental and Clinical Medicine, Division of Rheumatology AOUC; University of Florence, Florence, Italy (marco.matuccicerinic@unifi.it);

^3^Division of Rheumatology and Clinical Immunology, Department of Internal Medicine IV, Ludwig-Maximilians-University, Munich, Germany (Hendrik.Schulze-Koops@med.uni-muenchen.de);

^4^Leeds Institute of Rheumatic & Musculoskeletal Medicine, University of Leeds & NIHR Leeds Biomedical Research Centre, Leeds, United Kingdom (M.Buch@leeds.ac.uk);

^5^Medical University of Silesia, Katowice, Poland, (ejkucharz@poczta.onet.pl);

^6^Cochin Hospital, Rheumatology A department, Paris Descartes University, Paris, France (yannick.allanore@me.com);

^7^UC San Diego School of Medicine, La Jolla, California, USA (akavanaugh@ucsd.edu);

^8^Hexal AG, a Sandoz company, Holzkirchen, Germany, (philip.young@novartis.com) for Philip Young (goran.babic@sandoz.com) for Goran Babic

**Corresponding author:**

Janusz Jaworski,

Reumatika–Centrum Reumatologii,

02-691 Warsaw, Poland,

**Phone**: +48 602328612

**Email**: januszjaworski@ymail.com

**Table S1. Protocol deviations defined as major by category**

| **Category** | **Major protocol deviation** |
| --- | --- |
| **Selection criteria not met** | Deviations of inclusion or exclusion criteria that affected the study outcome with regard to the efficacy analysis |
| **GCP deviation** | No informed consent form signed; absence of three or more injections of study drug during a specific study period or of two or more consecutive injections at any time point during the study* |
| **Treatment deviation** | Patients who received incorrect study drug |
| **Prohibited concomitant medication** | Prohibited concomitant medication that may have impacted the efficacy endpoints |
| **Failure to perform key and safety procedure** | Missing components of the DAS28-CRP (CRP, tender or swollen joint assessments, patient global disease activity assessment) that may have an impact on the key efficacy analysis; Change, interruption or discontinuation of MTX dose; Visits used for the key efficacy analysis (Baseline, Visit 8 (Week 24) and Visit 12 (Week 48) DAS28-CRP measurement) were missing or outside of the visit window |

*Absence of one or two non-consecutive injections during the study was defined as a minor protocol deviation in this category.

CRP, C-reactive protein; DAS28, disease activity score including 28 joints; MTX, methotrexate.

**Table S2. HAQ-DI and FACIT-fatigue scores over 48 weeks (TP2 per-protocol set)**

| **Visits** | **Continued SDZ ETN**  **N=148** | **Switched to SDZ ETN**  **N=131** |
| --- | --- | --- |
| **HAQ-DI score*, Mean (SD)** | | |
| Baseline | 1.47 (0.55) | 1.50 (0.55) |
| Week 4 | 1.22 (0.55) | 1.14 (0.62) |
| Week 12 | 1.04 (0.56) | 0.97 (0.61) |
| Week 24 | 0.88 (0.59) | 0.83 (0.60) |
| Week 36 | 0.89 (0.58) | 0.85 (0.65) |
| Week 48 | 0.85 (0.61) | 0.84 (0.65) |
| **FACIT-fatigue score^†^, Mean (SD)** | | |
| Baseline | 26.6 (9.71) | 25.5 (10.78) |
| Week 4 | 31.5 (8.72) | 31.3 (10.07) |
| Week 12 | 34.4 (8.89) | 34.1 (9.69) |
| Week 24 | 36.6 (8.75) | 36.7 (9.05) |
| Week 36 | 36.9 (8.79) | 36.4 (9.50) |
| Week 48 | 38.0 (8.74) | 35.8 (9.97) |

*For HAQ-DI, the range of possible scores is 0–3 (0 being the best possible score and 3 the worst). **^†^**The FACIT-F score ranges from 0–52, with lower scores indicating more fatigue.

HAQ-DI, health assessment questionnaire disability index; FACIT, functional assessment of chronic illness therapy; SD, standard deviation

**Table S3. TEAEs of special interest (TP2 safety set)**

| **Preferred term** | **Continued SDZ ETN**  **N=175**  **n (%)** | **Switched to SDZ ETN**  **N=166**  **n (%)** |
| --- | --- | --- |
| Alanine aminotransferase increased | 4 (2.3) | 6 (3.6) |
| Transaminases increased | 3 (1.7) | 2 (1.2) |
| Aspartate aminotransferase increased | 3 (1.7) | 1 (0.6) |
| Hepatitis toxic | 1 (0.6) | 0 |
| Urticaria | 1 (0.6) | 0 |
| Hepatic enzyme increased | 0 | 3 (1.8) |
| Cardiac failure | 0 | 1 (0.6) |
| Cardiac failure chronic | 0 | 1 (0.6) |

TEAEs are events started after the first dose of study treatment and before study discontinuation or 30 days after last dose, whichever occurs later. A patient with multiple occurrences of event within the same system organ class or preferred term under one treatment is counted only once. Preferred terms are sorted in descending frequency, as reported in the “continued SDZ ETN” column.

**Table S4. Summary of anti-drug antibodies up to Week 48 using a 1% false positive cut-point (Safety set)**

| **Visits** | **Results** | **SDZ ETN**  **N=186**  **n/M (%)** | **ETN/SDZ ETN**  **N=190**  **n/M (%)** |
| --- | --- | --- | --- |
| Baseline | Negative | 184/186 (98.9%) | 190/190 (100%) |
|  | Positive | 2/186 (1.1%) | 0/190 |
|  | Neutralizing | 0/2 | 0/0 |
| Week 2 | Negative | 181/183 (98.9%) | 180/185 (97.3%) |
|  | Positive | 2/183 (1.1%) | 5/185 (2.7%) |
|  | Neutralizing | 0/2 | 0/5 |
| Week 4 | Negative | 180/183 (98.4%) | 143/185 (77.3%) |
|  | Positive | 3/183 (1.6%) | 42/185 (22.7%) |
|  | Neutralizing | 0/3 | 3/42 (7.1%) |
| Week 12 | Negative | 179/179 (100%) | 171/176 (97.2%) |
|  | Positive | 0/179 | 5/176 (2.8%) |
|  | Neutralizing | 0/0 | 1/5 (20%) |
| Week 24 | Negative | 179/179 (100%) | 172/172 (100%) |
|  | Positive | 0/179 | 0/172 |
|  | Neutralizing | 0/0 | 0/0 |
| Week 30 | Negative | 169/171 (98.8%) | 161/161 (100%) |
|  | Positive | 2/171 (1.2%) | 0/161 |
|  | Neutralizing | 0/2 | 0/0 |
| Week 36 | Negative | 169/169 (100%) | 159/159 (100) |
|  | Positive | 0/169 | 0/159 |
|  | Neutralizing | 0/0 | 0/0 |
| Week 48 | Negative | 164/166 (98.8%) | 154/154 (100%) |
|  | Positive | 2/166 (1.2%) | 0/154 |
|  | Neutralizing | 0/2 | 0/0 |

M, number of patients with evaluable data; n, number of patients per treatment group with anti-drug antibodies result. Patients with positive baseline anti-drug antibody responses are not included in post-baseline anti-drug antibody analysis (n and M).

ETN, reference etanercept; SDZ ETN, Sandoz etanercept
